# Feasibility analysis of novel Maglev EDM by comparing with conventional micro EDM

**DOI:** 10.1038/s41598-022-06662-1

**Published:** 2022-02-16

**Authors:** Mangal Singh Sisodiya, Shashank Shukla, Vivek Bajpai

**Affiliations:** Department of Mechanical Engineering, Indian Institute of Technology (ISM), Dhanbad, Dhanbad, India

**Keywords:** Engineering, Physics

## Abstract

EDM is the most popular unconventional machining process. The present technology of EDM consists of a pulsed or capacitive type power supply in which the pulsed type power supply is more popular and effective. The following essential component of an EDM is its servo mechanism, which controls the gap between the electrodes and maintains the gap voltage. A low machining speed, complex power supply, and servo mechanism increase the cost of machining and the maintenance cost of an EDM machine. To resolve the above issues from the EDM, a novel servo mechanism has been developed, which is simple in design and low in cost and has the capacity to use direct current as a power source. The current work elaborates a brief description of the novel servo mechanism and its feasibility analysis. Pure DC power is employed with the conjunction of Maglev lucidity to refine the shortcomings of conventional micro EDM. The novel technology addresses the prime concerns of conventional micro EDMs and deficiencies such as the delayed response of mechanical actuators and a servo mechanism. The novel technology uses the logical arrangement of permanent magnets and electromagnets to address inadequacies such as short circuiting and arcing. The work outlook is to establish the viability of the novel Maglev EDM by a comparison with a similar range of parameters. The results on the novel technology showed an improved material removal rate (MRR), which was in the range of 76.6 μgm/min, whereas the specific energy and surface roughness were 33.4 Joule/microgram and 4.3 μm, respectively, while machining commercially pure titanium.

## Introduction

An electrical discharge machine (EDM) is the most popular and appropriate unconventional machining technique for machining hard-to-cut materials such as titanium and nickel-based alloys. Although superalloys are useful in advanced areas, their low thermal conductivity and high strength make them difficult to cut. Therefore, processing such materials by conventional machining methods is almost impossible or not worthwhile economically. Hence, the applications of nonconventional or advanced machining methods such as EDM have become popular. Owing to the practicality of EDM, the process has extended up to the micro level, i.e., micro-EDM. Microscopic EDM has become a useful technique for producing microfeatures in hard materials. Micro EDM differs from conventional EDM in terms of the size of the electrode tool, low amplitude of the energy pulse, and high resolution to achieve fine finishing. The pulse generator generates micro EDM minute pulses for a few microseconds or nanoseconds. As a result, a small volume of work material is removed, ranging from 0.05 to 500 μm^3^, because the low energy ranges from 10 ^−9^ to 10 ^−5^ J. The machining of difficult-to-cut materials, such as titanium alloys, through micro EDM exhibits issues such as a lower material removal rate (MRR), high tool wear, generation of a recast layer, and improper flushing of debris^1^. In general, the material removal rate (MRR) of the micro-EDM lies in the range of 0.6 mm^3^/h to 6.0 mm^3^/h, depending on the process parameters^2^. However, such a low material removal rate is not economical; hence, there is a research scope to improve the performance by modifying the micro-EDM system. EDM is an established machining technique, although the performance enhancements are still dubious. Its dependence on many input parameters makes the process stochastic^3^. In the past, several attempts have been made to improve the MRR through various techniques. Some researchers have used a magnetic field near the machining zone to improve the MRR by flushing out the debris. The researchers compared machining characteristics by developing magnetic assistance techniques. In one study, the effect of magnetic force assistance was analyzed by observing waveforms of current and voltage, and it was found that a magnetic field is helpful to increase the capability of the EDM process^4^. The magnetic field assistance further enhanced by employing different flushing methodologies results in a higher MRR in conventional EDM. However, it has been reported that size limitations and fragile tool materials restrict peripheral flushing in micro-EDM. It is also observed that magnetic assistance is favorable for higher MRRs only, with no significant effect on the surface roughness. On the other hand, magnetic assistance increases the tool wear rate (TWR) by increasing the distortion^5^. The literature shows that the MRR is not only the single process output of EDM; parameters such as TWR and surface roughness (SR) also illustrate the capability of micro-EDM. Similar to conventional EDM, the performance of the micro EDM can also be evaluated on the output response, which is mainly associated with the input process parameters. The input parameters include the discharge current, discharge voltage, pulse on time, pulse off time, duty factors, dielectric medium, and electrode gap between the tool and workpiece. The researcher studied the effect of various input parameters and identified that the discharge current was the most influencing factor, which affects the process efficiency overall. In the early stage of EDM, the relaxation-type pulse generator was in use with capacitor discharge. The applications of relaxation types of pulse generators are common in wire EDM (WEDM). Such a type of pulse generator has provided a high peak current for a very short time, which is a favorable condition for a higher MRR^6^. Later, the relaxation-type generator was replaced by a transistor-type generator, with an improvement in power transistors. Transistor-type generators are applicable, where a large amount of current with a high response is required. However, relaxation-type generators are still in use on micro EDM and WEDM. Relaxation-type generators are capable of generating short pulses with constant energy pulses, although the time taken in capacitance recharging is the drawback of relaxation generators. Over time, pulse generation apparatuses and tools have been developed by inventors, although none of them give adequate efficiency to the process. Since it has been observed that an increase in the pulse duration improves the MRR to some extent, a longer pulse lowers the MRR due to a reduction in the plasma strength^7^. Hence, moderate pulse generation is essential to achieving optimum results. Overall, stable discharge is essential via the utilization of each energy pulse to obtain a higher MRR, low tool wear, and high-quality surface. Furthermore, it has been observed through research that a precise apparatus is required to provide appropriate gap conditions between electrodes, which ensure effectual sparking during erosion^8^. To execute the toned gap condition, study of the existing feed mechanism is required. In the present work, the authors have reviewed the available literature and drawn succeeding inferences. The study emphasizes that the electrode gap, known as the actual spark gap, is maintained using a servo control mechanism in conventional EDM. The spark gap plays an important role and is solemnly responsible for achieving efficient machining in terms of higher MRR and low surface roughness (SR) with minimum TWR. It is well known that the actual EDM processing is conducted in a narrow space (usually in microns) between two electrodes, with one cutting tool and a workpiece. An adequate gap between the electrodes is essential to maintaining efficient machining in EDM. A higher electrode gap reduces the plasma strength. However, the smaller electrode gap hinders effective deionization at the time of pulse rest. Improper deionization leads to harmful effects, such as short circuiting, which affects the surface quality of the workpiece surface. Especially in micro EDM, the metal is eroded in the form of micro- and nano-size debris and eventually accumulates in a narrow space between the electrodes. The accumulation of eroded material particles in the narrow space increases the chance of inefficient discharge energy, ultimately reducing the efficiency of micro EDM. Referring to the above importance of the process and maintaining the electrode gap using a servo mechanism, maintenance or optimization is required. The use of conventional motors and ball screws in servo mechanisms reduces the capability of micro-EDMs due to their slower response. Efforts have been made by researchers to improve the meticulous electrode gap using magnetic actuators. Every pulse is important for speeding up the machining by keeping the appropriate electrode gap; however, the conventional servo mechanism is not capable of regulating the proper gap^9^. Previous research has been conducted to improve the efficiency of micro EDMs by incorporating two-stage fuzzy logic controllers. In this system, the first stage is used for the detection of the discharge, and the second stage has high precision and is used as the servo stage for precise control over the discharge gap^10^. In general, an RC-type power supply was used in the micro-EDM process. The RC power supply must continue the motion of the servo system up and down to help the charging and discharging of the capacitor. This movement reduces the material removal and hence introduces another vibrating actuator. A vibrating tool was introduced to assist the up-down motion of the stage, which helped to improve the charging-discharging cycles and hence improved the MRR^11^. An advanced version of the vibrating tool electrode was introduced by Zhang et al.^12,13^. An improved system was developed with the magnetic actuator. The magnetic actuator was levitated on magnetic hearing, and motion was controlled by the voice coil electric magnet. A PID (proportional-integral-derivative) controller was used to control the tool’s electrode tip at a very low magnitude of vibration (approximately 2 mm). The work was further improved by the same research group, and a high material removal rate with stable discharge was reported^14,15^. Although magnetic levitation was successfully reported with the RC power supply, it was an add-on to the existing EDM machine. A demonstration of the tension and bending of the particle raft has been reported^16^, which has motivated the control of the magnetic actuator more precisely and is dependent on the electrode gap voltage. Furthermore, the pulse power circuit and servo mechanism increase the intricacy of the system, which leads to high upkeep costs and energy in EDM. The present study is conducted to address the above prominent issues by developing novel Maglev EDM technology. Although Zhang proposed the maglev actuator and successfully implemented it in an existing EDM, the proposed maglev EDM differs from that system. The proposed system does not require an EDM machine, and the second major feature of the proposed system is its control. The circuit is designed in such a manner that the current in to the electric magnet will be governed by the electrode gap. It is an adaptive system and hence can use a DC power supply for EDM instead of an RC power supply or pulsed power supply. To validate the results and feasibility of the system, a series of experiments was performed. The experimental results demonstrate the improved performance of the Maglev micro EDM, which was significantly improved over the existing EDM technology. The characterization of MRR and SR pointed out the capabilities of the novel Maglev EDM. A detailed explanation and comparison are reported in the following sections.

## EDM working principle

**Figure 1:**
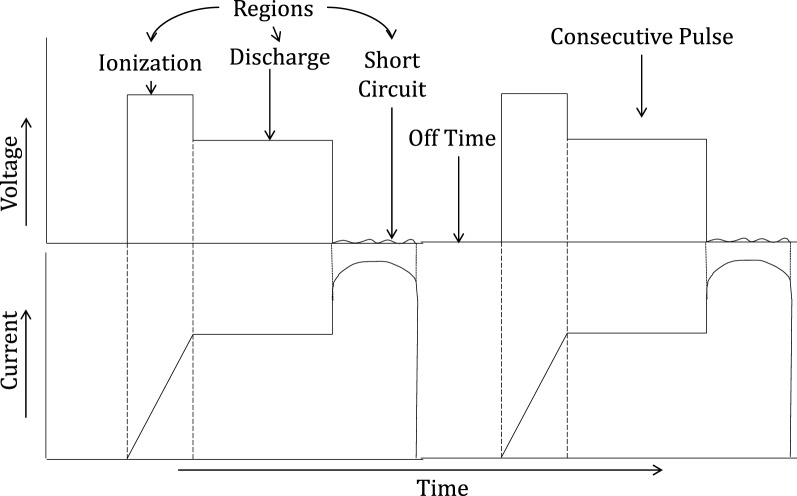
Voltage and current graph of the conventional EDM process^22^.

EDM is based on the generation of potential differences between the two electrodes, namely, the cutting tool and the workpiece. In EDM workpiece material eroded by the sequential reoccurrence of an electric spark between the two electrodes, the cutting tool acts as the cathode and the workpiece acts as the anode^17^. The dielectric medium kerosene or deionized water is used to achieve a discharge between the electrodes, the proper flushing of debris and avoidance of thermal stresses via extremely high temperature^18^. During the process, an appropriate range of voltages is supplied to the electrodes. The narrow gap, usually 0.005–0.05 mm between the tool and the workpiece, is maintained by the servomechanism. Owing to the establishment of an electrical arena in the narrow gap between electrodes, the free electrons are subjected to electrostatic force and start to move from the cathode (tool) to the anode (workpiece) via the dielectric medium. These free electrons gain energy and strike the molecules in the dielectric fluids. Such an impact results in the ionization of dielectric fluid. When the ionization is in its peak, a sharp plasma channel is generated, which has a very low electrical resistance. Consequently, a large number of electrons collide with positive ions. This phenomenon produces a large amount of local intense heat. Thus, through intense local heat, the material starts to vaporize and melt from the workpiece as well as from the tool. Accordingly, a negative impression of the cutting tool is produced in the form of a cavity on the workpiece without physical contact between the tool and work. Since contact-free metal removals from the workpiece occur in EDM, the process is considered to be one of vibrational chatter, and mechanical stress-free machining techniques^19^. In the EDM process, the temperature between the tool and workpiece is mainly a function of the power supply and the narrow gap between the electrodes. Therefore, the EDM performance primarily depends on these important factors. The influencing parameters in EDM can be categorized in two ways: electrical parameters such as discharge current, discharge voltage and pulse on time and nonelectrical parameters such as electrode gap, flushing medium, and tool life ^20^. The researcher investigated EDM in all aspects and specified managing all influencing parameters to improve the machining efficiency^21^. They suggested various techniques, such as ultrasonic vibration, dry EDM, and powder EDM. In conventional EDM, each process parameter is precisely controlled for the optimum response output. In EDM, pulse generation and duration are continued by power regulatory devices. Each energy pulse can be categorized into three subsequent zones: ionization, discharge, and short circuit zone, as shown in Fig. 1. During the machining process, the first ionization of the dielectric fluid is conducted, which helps to increase the discharge energy; consequently, a high required spark is produced in the second discharge zone for material removal. The third short circuit is an unwanted zone and needs to be avoided in machining by interrupting the power supply for a small time and then switching it on to obtain another stable spark. The short-circuiting phenomenon is harmful to the quality of the EDM surface; hence, it has been eliminated in the present work to a great extent by employing novel EDM technology. The proficiency of the novel machine is presented in the following section.

## Experimental methodology

**Figure 2:**
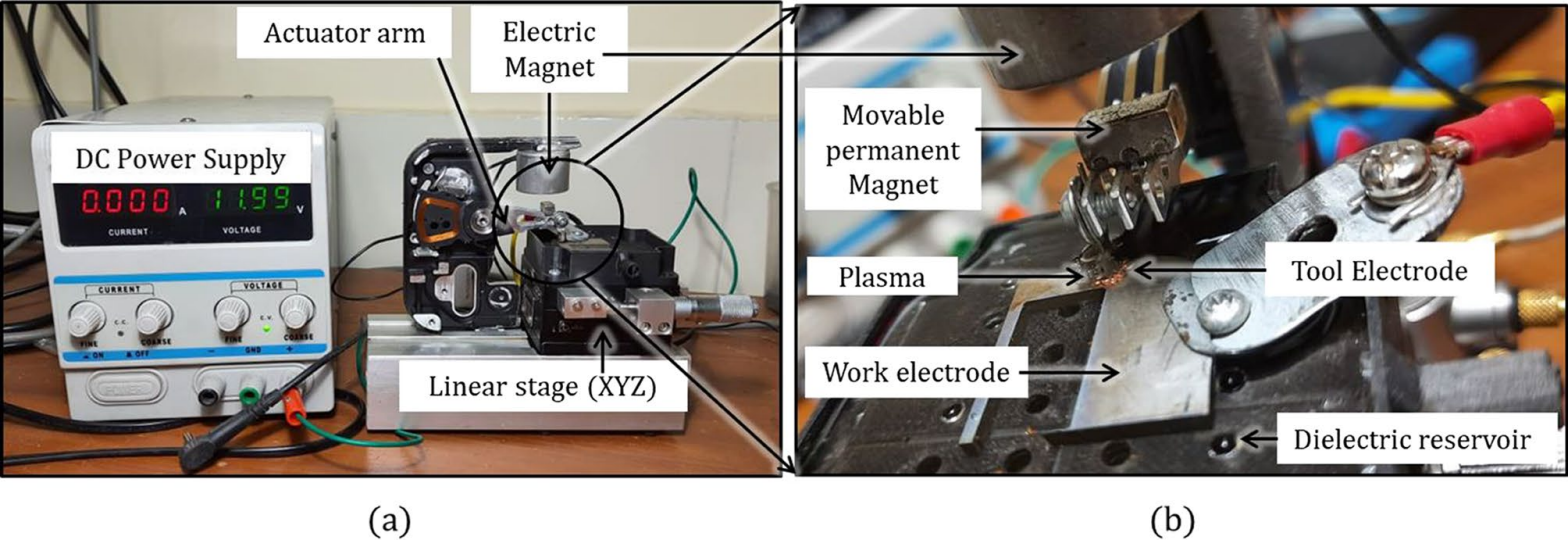
(**a**) True scale working prototype of Maglev EDM; (**b**) magnified view of machining zone.

**Figure 3:**
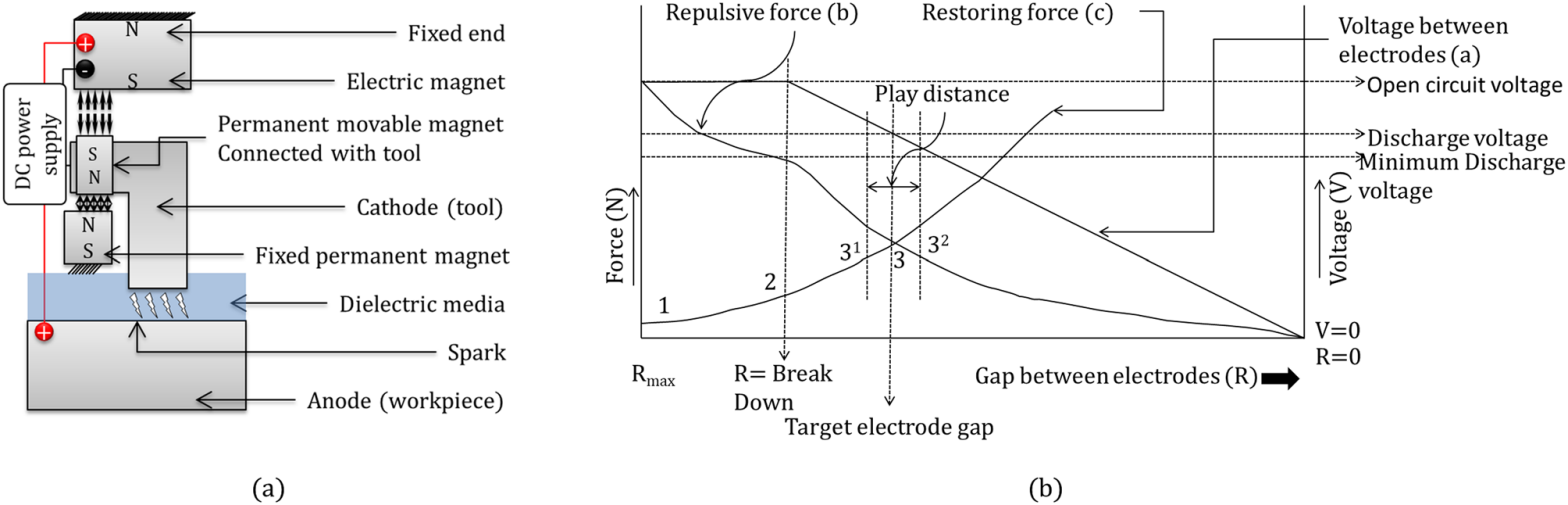
(**a**) Schematic view of the arrangement of the components; (**b**) graphical representation of tool movement in Maglev EDM.

A true-scale working prototype was developed (Fig. 2a) with an enlarged view of the machining zone (Fig. 2b). Discharge is produced between the electrodes by maintaining a specified gap between them. In the current technology, discharge is produced by maintaining a specified gap between the electrodes. The proposed technology has an arrangement of magnetic levitation to maintain the electrode gap. A schematic diagram of the working maglev EDM is shown in Fig. 3a. It consists of two sets of magnetic repulsive forces. One set consists of a pair of electric magnets and a permanent moving magnet. The tool is fixed on the moving magnet and can travel in such a way that the gap between the tool and the workpiece can be adjusted. The other magnetic pair consists of two permanent magnets: one is fixed, and the other is the same moving permanent magnet that has a tool on it. The fixed magnet pushes the tool away from the workpiece, and the electric magnet pushes the tool toward the workpiece. Figure 2a, b show only one magnetic pair pushing the tool toward the workpiece. The other pair is behind the actuator arm and is not visible in the figure.

A DC power supply is connected to the electric magnet and the electrodes in parallel. This magnetic field produces a repulsive force on the movable permanent magnet (connected to the tool), which results in the movement of the electrode tool toward the workpiece electrode. This movement of the tool reduces the gap between the electrodes, and after a threshold gap between the electrodes, ionization followed by electrical discharge occurs. The discharge reduces the voltage between the electrodes, and since the electrodes are connected in parallel with the electric magnet, the voltage across the electric magnet decreases. Consequently, the strength of the electric magnet is reduced. Meanwhile, the distance between the other pair of permanent magnets decreases, and hence, the restoring repulsive forces increase. The variation in the two repulsive forces and their balancing maintains the electrode gap, as illustrated in detail in Fig. 3b. In this figure, there are three labeled curves, which are as follows:

- Curve—a: Variation in the voltage applied between the electrodes and electric magnet.
- Curve—b: The repulsive force by the electric magnet to the moving permanent magnet plus tool, which reduces the electrode gap
- Curve—c: The restoring repulsive force by the fixed permanent magnet to the moving permanent magnets plus the tool, which increases the electrode gap.

At the start of the process, when the electrode tool is at the home position, there is an extreme gap between the tool and the workpiece. When voltage is applied to the system, the electric magnet has the maximum voltage, and the distance between the electric magnet and the movable magnet is minimal. Hence, the repulsive force is at its highest value. The repulsive magnetic force is represented mathematically by the following relation:

1 $${\varvec{Force }} \propto \frac{{\left( {{\varvec{m}}_{1} \times {\varvec{m}}_{2} } \right)}}{{{\varvec{r}}^{{2}} }}$$

In the above relation, m_1_ and m_2_ are the strengths of the magnets, and r is the distance between the magnets. As the moving magnet moves the tool toward the workpiece, the distance between the electric magnet and the moving permanent magnet increases, and hence, the repulsive force decreases (Point 1 to point 2 in Fig. 3b), and at the same time, the distance between the restoring pair of magnets decreases; hence, the restoring force increases. Point 2 represents the threshold value of the gap voltage; hence, after this point, not only will the distance between the electric magnet and the movable magnet increase but the voltage in the electric magnet will also decrease. Hence, the repulsive force will decrease more rapidly after point 2. Starting from point 1 and passing point 2, the repulsive restoring force increases due to the reduction in the distance between magnets. In Fig. 3b, point 3 is the location where the repulsive force and restoring force are equal and the tool electrode stays to deliver the required discharge for machining. However, the tool oscillates near point 3 due to inertia, which results in an unbalanced condition in the repulsive force and restoring force. In Fig. 3b, the oscillation path is shown by the play distance. In the current work, the electrode gap and the movement of the tool electrode are precisely maintained at a play distance to generate optimum discharge energy using maglev lucidity. At present, the play distance has a finite value but does not hamper the discharge, as described by the V-I characteristics shown in the next sections. It is expected that the play distance can be minimized by applying mini/micro dampers to the tool motion.

In the present work, experiments were performed on commercially pure titanium CP-TI. A small plate with a thickness of 3 mm and a polished surface was used to ensure parallelism on the stage. CP-TI is the most commonly used material for making nonstructural applications, such as water channels, ducts, and piping, owing to its special characteristics of high strength at elevated temperatures, corrosive resistance, and good formability. A brass tool electrode with a 2 mm diameter was used with deionized water as a dielectric medium. Both electrodes are connected to a direct current (DC) power supply in such a manner that the cutting tool and workpiece act as the cathode and anode, respectively. The specific resistance of the work reduces in positive polarity, which helps in enhanced MRR^23^. In the present work, a 12 V open-circuit voltage and a 2 amp peak current are applied through the DC power supply. A digital oscilloscope (Tektronix, TDS2012C, 2-channel, 100 MHz bandwidth) using a differential type voltage (TPP0201, Tektronix) and current probe (65A-Hantek, BNC type) was used to assess the waveforms of the discharge voltage and current. The workpiece material is eroded in the shape of the circular pocket with a 2 mm diameter in four different experiments. The experiments were repeated three times to minimize experimental errors. A digital weighing pan (Mettler Toledo) with a minimum count of 10 μg was used to measure the weight of the workpiece before and after machining.

## Results and discussion

The experiments were conducted systemically, and all of the observations were noted sensibly. In the following sections, the explanation and science behind the occurrences of all response outputs are discussed. The entire process itself adequately explained and validated the EDM. In the process, metal is removed from the harder workpiece (CP-TI) without any physical contact with the tool (brass), which is a comparatively soft material. To demonstrate the process, the voltage-current plot of maglev EDM was explained and compared with the existing die sink EDM machine. The material removal rate was analyzed and compared with the existing literature. The specific energy of the machining is a measure of the process efficiency. Low specific energy shows high machining efficiency. The specific energy of the machining is calculated for the maglev EDM and compared with the existing literature, which showed that the specific energy is minimum in maglev EDM for CP-Ti. The other key response in an EDM process is the surface roughness. The following sections provide insights into the capabilities and proficiency of the novel Maglev EDM.

### Discharge voltage current characteristics

**Figure 4:**
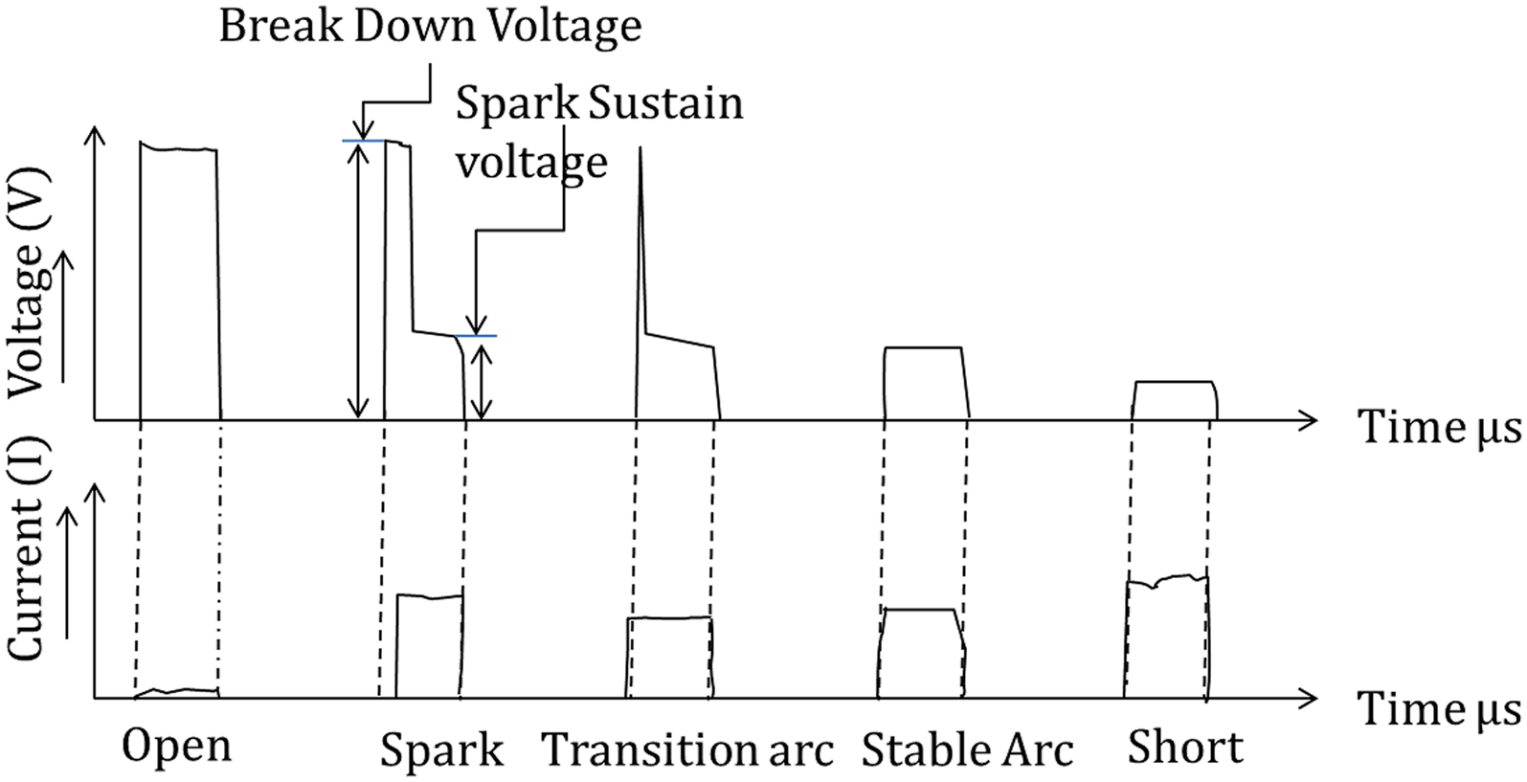
The five different possible EDM conditions^25^.

The discharge voltage and current characteristics play a crucial role in the formation of the required appropriate spark between the electrodes. In general, during EDM, there can be five different types/conditions of pulses, as shown in Fig. 4. These all are based on the input process variables. In the first type, open voltage is the condition where the voltage will be maximum and the current will be zero. This circumstance is the ideal condition in which there is no removal of work material. The next condition is that spark or normal spark conditions have sufficient capability to perform proper machining that utilizes high current amplitudes between electrodes. The normal spark provides the proper delay for the breakdown and recovery of the dielectric strength of the liquid medium for the next pulse. The transition arc or stable arc is a common phenomenon except that the stable arc has no high-frequency component as in a transition arc. However, the stable arc differs from the normal discharge and has a very weak high-frequency signal in contrast with normal discharge. In the last possible phenomenon, short circuiting is the state in which the gap voltage is almost zero and the current amplitude is the highest. Short circuiting occurs for various reasons, such as debris that formed between electrode gaps, very low electrode gaps, or physical contact of electrodes^24^. Short circuiting affects the process stability and leads to a lower MRR with a low quality machined surface.

**Figure 5:**
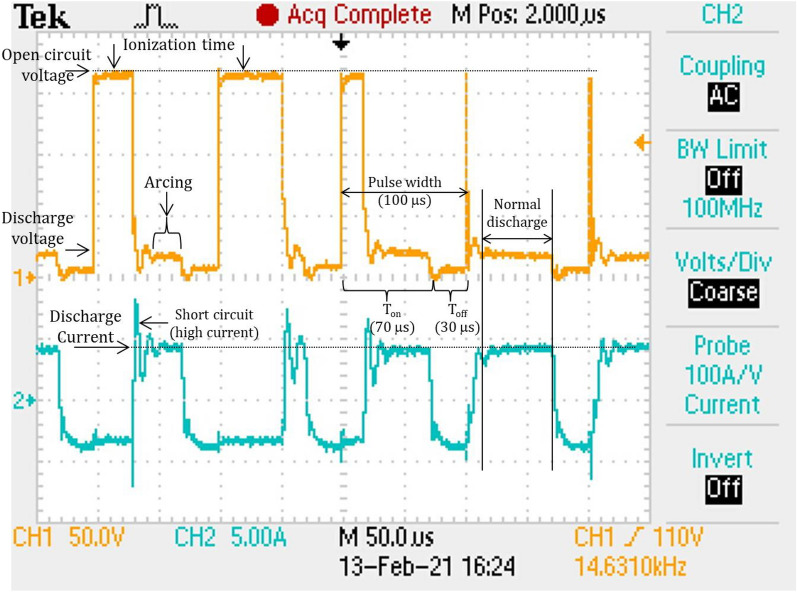
Illustration of the discharge waveform of conventional EDM.

Further investigation of the voltage-current waveform found that arcing is another harmful phenomenon for the quality of the surface in EDM and micro EDM. The arcing is the result of the uncontrolled transformation of thermal energy during the process. Arcing is the successful discharge of current before attaining the open-circuit voltage, which results in repetitive discharge at the same location^26^. During arcing, the current flows in the same plasma without recharging the capacitor and recovering the dielectric strength of the previous discharge^27^. To elaborate on the above conditions, experiments were performed on a conventional die sink EDM (SPARKONIX ZNC/ENC35). Figure 5 shows the voltage-current characteristics recorded during conventional EDM. The plot shows arcing, short circuit, ionization, and normal discharge during conventional EDM.

In the study of waveforms, it is observed that an ignition delay occurs as the voltage is applied between the electrodes, and the dielectric does not break down immediately, which results in a small static time lag. The researcher investigated the spreading of the ignition delay and determined that discharge occurred after the activation time^28^. To attain a stable spark, the discharge interval should be sufficiently long to enable proper plasma and dielectric strength to be generated and recovered, respectively. However, it has been reported that an interval that is too long leads to a low MRR^6^. Hence, the precise control of electrode movement is the most essential, especially in micro EDM.

In addition to the discharge interval, the plugging of debris in the electrode gap, the electrode geometry, and inappropriate input parameters affect the machining with regard to arcing and short circuiting (which is the most undesirable). The steady discharge energy is the prime requirement for micro EDMs.

**Figure 6:**
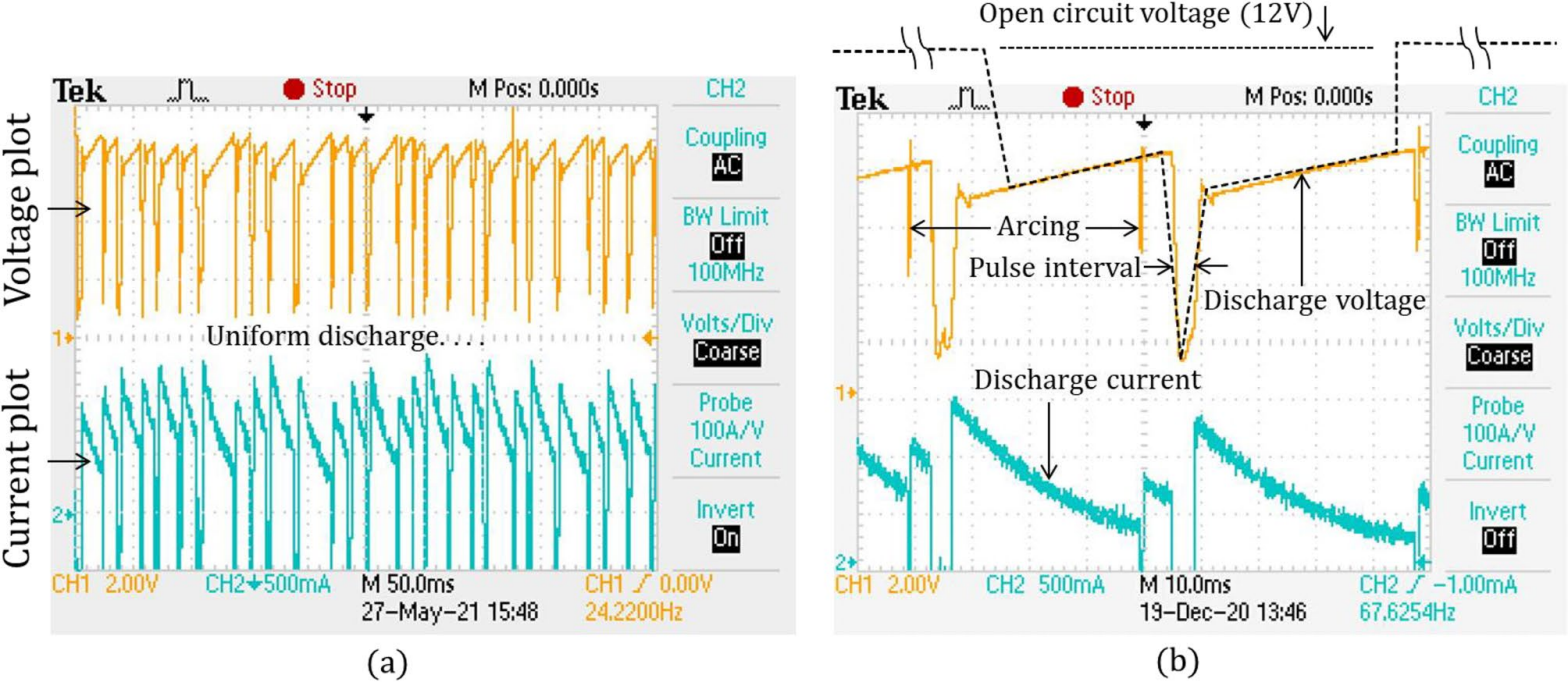
(**a**) Voltage and current characteristics showing stable and uniform discharge; (**b**) detailed pulse characteristics.

Hence, the attention and consideration of voltage-current waveforms are the most important in the process. In the current work, a digital storage oscilloscope (DSO) is utilized to observe the waveform of the discharge voltage and current. Figure 6a, b illustrate the discharge voltage and current waveforms of the Maglev EDM. The waveforms clearly express that the discharge is stable throughout the process. Since the power supply is a pure DC power source, the Toff time is zero in the waveform. Figure 6a shows multiple pulses, which show a uniform discharge after each retraction of the tool. Figure 6b shows two pulses in detail. No short circuit is shown in the waveform, which is nearly impossible in conventional EDM. Almost stable discharge with a negligible arcing at a few places is shown. The proper discharge without short circuiting ensures a higher MRR in Maglev EDM. No ionization time is visible in the plot due to zero off time in the process. This finding again supports a high energy supply and less nonproductive time and enhances the MRR.

Overall, the Maglev EDM proceeds in a continuous manner without any abruption, as occurred in conventional EDM, which indicates the proficiency of the Maglev EDM.

### Material removal rate (MRR)

The MRR and TWR are the most significant factors in the machining process. These factors merely affect the productivity of the manufacturing firms over time. The MRR is the weight difference of the workpiece before and after machining, divided by the machining time. Similarly, TWR is the difference in the weight of the tool before and after machining and divided by the machining time.

2 $$MRR = {\raise0.7ex\hbox{${\left( {w_{b} - w_{a} } \right)}$} \!\mathord{\left/ {\vphantom {{\left( {w_{b} - w_{a} } \right)} T}}\right.\kern-\nulldelimiterspace} \!\lower0.7ex\hbox{$T$}}$$

3 $$TWR = {\raise0.7ex\hbox{${\left( {T_{b} - T_{a} } \right)}$} \!\mathord{\left/ {\vphantom {{\left( {T_{b} - T_{a} } \right)} T}}\right.\kern-\nulldelimiterspace} \!\lower0.7ex\hbox{$T$}}$$

where w_b_ and w_a_ are the weights of the workpiece before and after machining, respectively, and T is the machine time. T_b_ and T_a_ are the weights of the cutting tool before and after machining, respectively.

**Figure 7:**
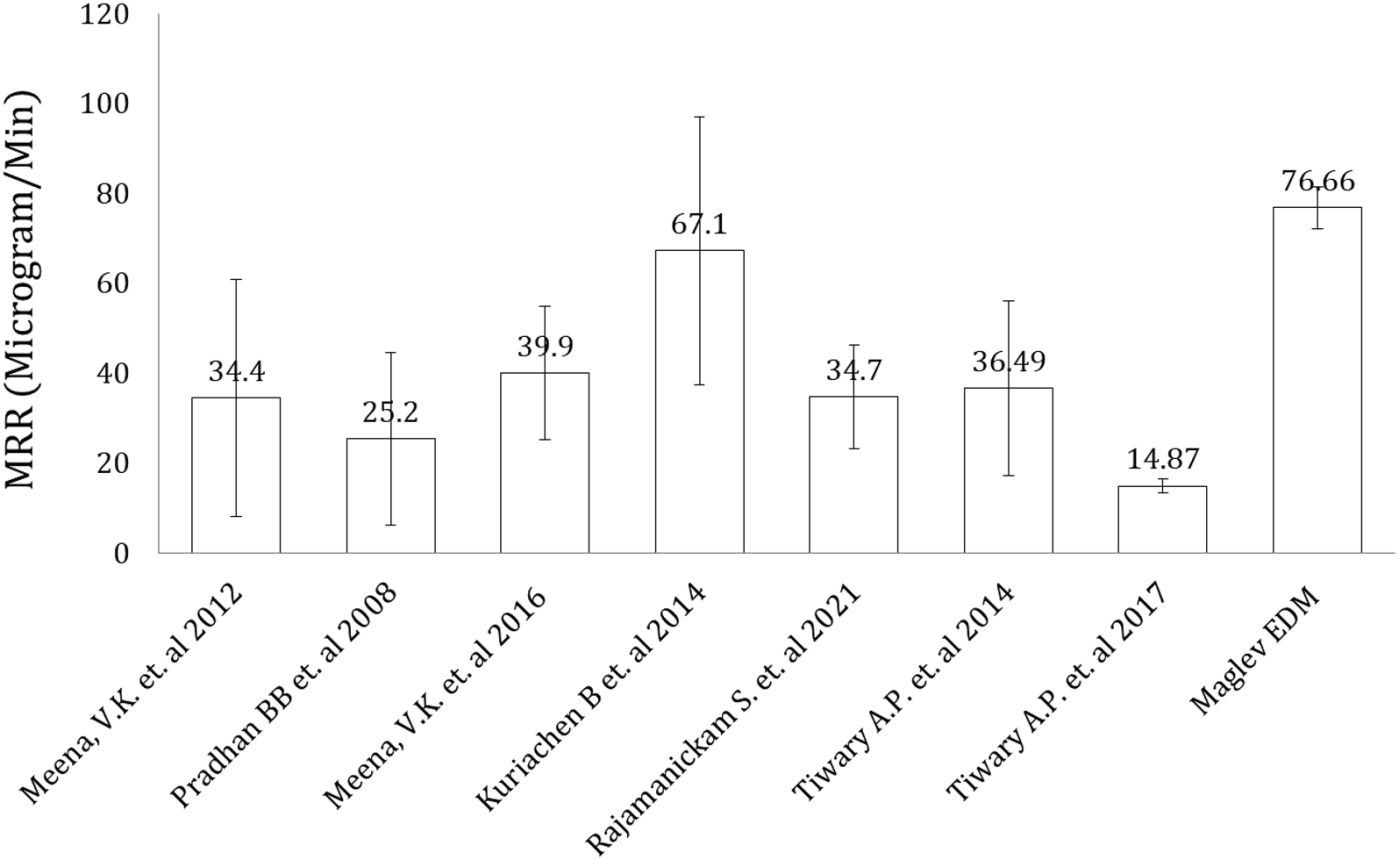
Comparison of the material removal rate between maglev EDM and the literature.

The MRR is the key component to be considered per the economic point of view of any machining operation. Similarly, in the field of EDM, especially in the machining of hard-to-cut materials, MRR is always a prime concern for researchers. From inspection to the present era, research continues to optimize this machining response. As per Fig. 7, many researchers have suggested that the tools and techniques enhance the EDM from time to time. The study by Meena et al.^29^ calculated the MRR at an average of 34.4 μg/min and concluded that the voltage is the most influential parameter among all of the parameters. The MRR observed by Pradhan et al.^30^ was in the range of 25.2 μg/min. They produced microholes in titanium alloy and observed that the pulse on time is the most significant factor in MRR and that the peak current mainly affects the TWR in micro EDM. They attributed the peak current to a higher MRR and high tool wear because of the monotonic increase in the high energy density. Later, Meena et al. confirmed that the current is the most promising factor among all of the parameters of micro EDM for higher MRR. They observed the MRR in the average range of 39.9 μg/min while machining commercially pure titanium. The MRR measured by Kuriachen et al.^31^ was in the average range of 67.1 μg/min during the multiresponse optimization technique. They used multiobjective particle swarm optimization and considered that the gap voltage influences the MRR in micro EDMs. Rajamanickam et al.^32^ calculated the MRR in the average range of 34.7 μg/min. They used tap water as a dielectric medium with and without additives for a comparison of conductive and nonconductive additives. Tiwary et al.^33^ observed the MRR in the range of 36.49 μg/min and concluded that the pulse on time, the peak current, and the gap voltage affect the range of MRR. During maglev EDM, the average measured material removal rate is 76.6 μgm/min, which is significantly higher than that of other conventional micro-EDMs for a similar range of discharge energy. In the current work, the maximum TWR was observed at 8.5 μg/min, the minimum TWR was noted at 2 μg/min, and the average TWR was found to be ~ 10% of the MRR in maglev EDM. The accumulation of eroded material on the tool electrode and the absence of pressure flushing are the largest causes of variation in the tool wear rate. The above results demonstrated the higher performance of Maglev EDM due to the availability of continuous discharge energy with a very low pulse off time compared to conventional micro EDM. In the current work, the values of MRR and TWR are observed at the natural flushing, and they can be additionally enhanced by employing a forced flushing system. Figure 7 shows the comparison of MRR attained by conventional EDM and attained with Maglev EDM. The MRR is higher, showing the capability of Maglev EDM over the other conventional EDM systems. The Maglev EDM shows a higher MRR because of the superior control of the interelectrode gap (IEG), which almost completely removes harmful effects such as short circuiting and arcing.

### Specific energy in EDM

In the previous section, a higher MRR is achieved in maglev EDMs compared to the literature; however, a higher MRR could be due to the high energy supply. The comparison could be fairer if specific energy could be compared with the available literature. It could be helpful to explore a wide range of literature with a dissimilar range of input process parameters. The specific energy is termed the energy required to remove the unit amount of material from the workpiece. It is denoted by SE and refers to EDM as in other machining processes. Specific energy is another factor that is crucial to defining the machining productivity of any manufacturing firm. In EDM, the SE is defined as follows:

4 $$SE = \frac{{\left( {Discharge\,power} \right)}}{MRR}$$

Furthermore, here, the discharge power can be evaluated in EDM as a product of the discharge voltage, current and duty factor.

**Figure 8:**
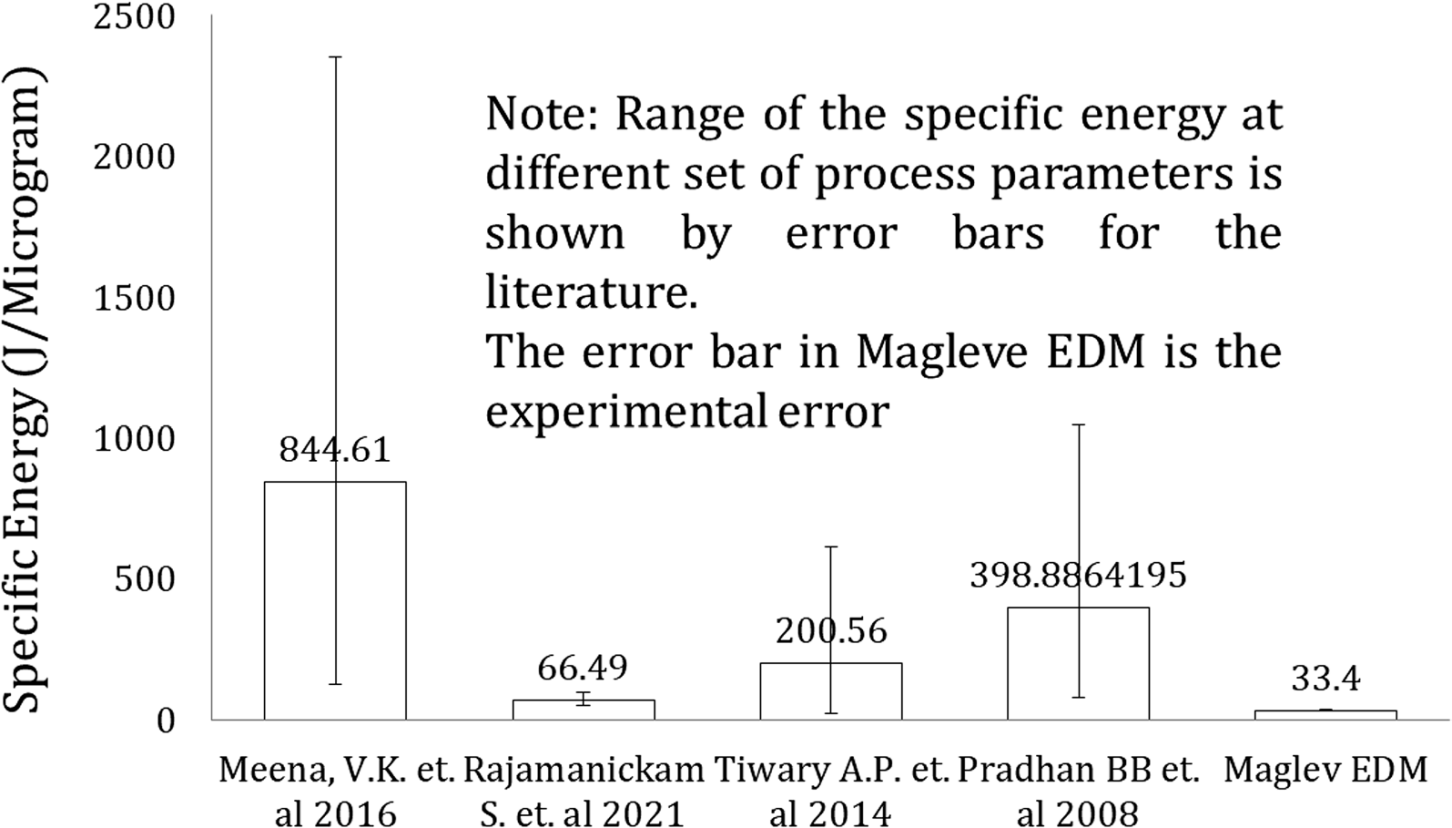
Comparison of the specific energy between the maglev EDM and the literature.

During the Maglev EDM, the maximum MRR was achieved at approximately 82 μg/min, and the SE ranged from approximately 30.76 to 35.21 J/g in three repetitions. A proper IEG leads to an efficient specific energy (SE) by utilizing each power pulse during spark generation. The specific energy of past reported work with Maglev EDM has been studied and presented in Fig. 8. From the comparison plot, the specific energy is found to be lowest in the Maglev EDM for the same range of MRRs compared to the literature. This finding indicates that the current Maglev EDM utilizes low energy to erode the significant material, which demonstrates the proficiency of the current Maglev EDM over conventional EDM.

### Surface morphology and surface roughness

**Figure 9:**
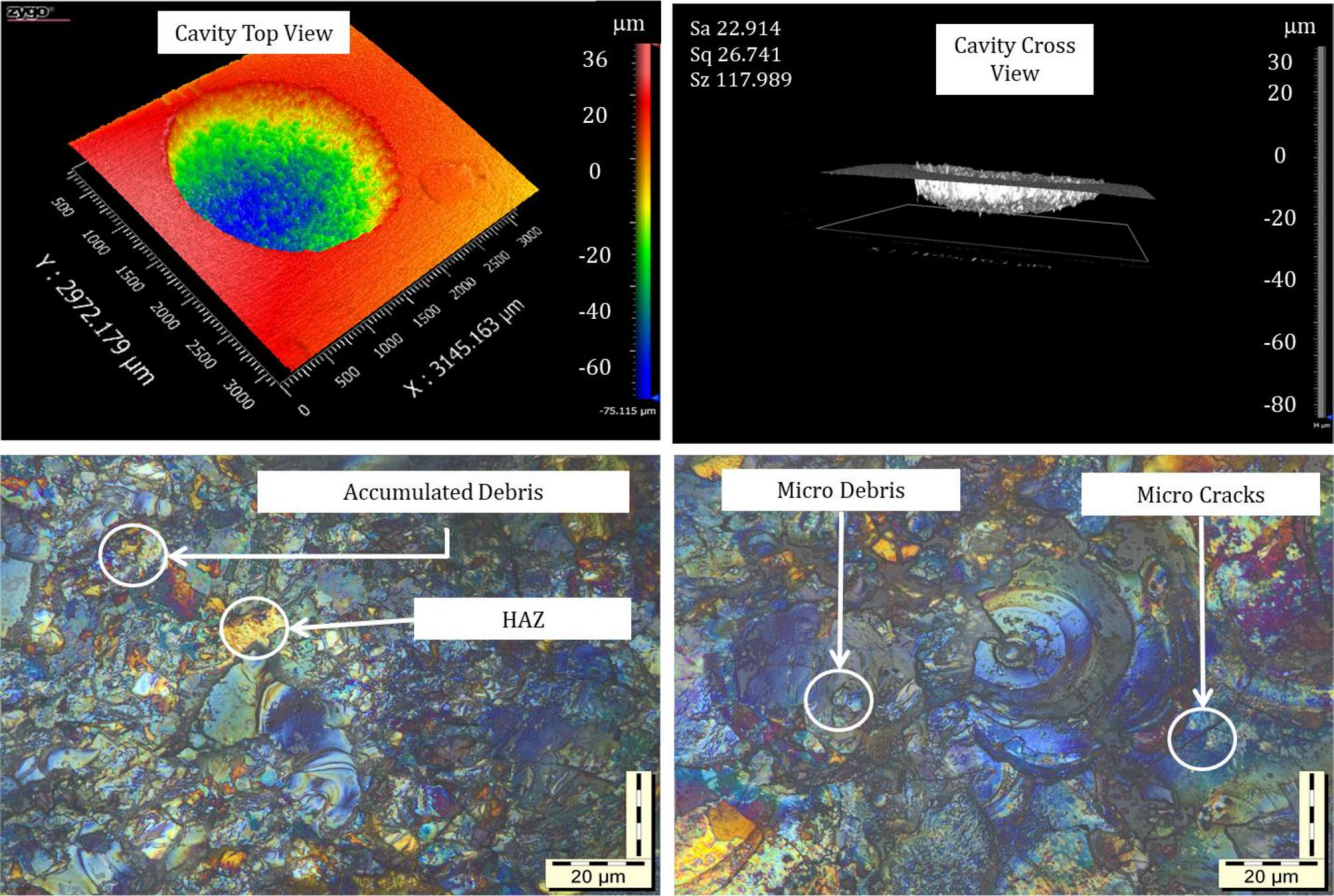
Optical microscope and profilometer images of surface morphology.

The surface characteristics of EDM components can be demonstrated by the arbitrary range of craters and hills, which are formed through discrete discharge pulses. The machining parameters significantly affect the level of surface roughness by resulting in overlapping craters and hills. An excessive level of surface roughness is undesirable in engineering applications such as in the molds and dies manufacturing industries^34^. The recast layer is one of the downsides of EDM and is a prime issue to be considered in the enhancement of surface quality^35^. The recast layer was developed by the lack of appropriate elimination of melted material and resolidification again, similar to quenching phenomena^36^. The recast layer is closely associated with the pulse duration. The long pulse duration can reduce the thickness of the recast layer to a great extent. Furthermore, the thickness of the recast layer depends on the type of dielectric, as a study reported that kerosene dialectics form a thinner layer compared to other dielectric media^37^. The long pulse releases more energy to enhance the effect of the dielectric force and to reduce the thickness of the layer further^38^. It has been seen that the surface roughness values of the machined component are increased at higher currents as well as during longer pulse durations^39^. Since the discharge energy is associated with the melting and vaporization of the workpiece material, with high discharge energy, larger craters are produced on the work surface, which deteriorates the quality of the surface. In some instances, the process is further prolonged undesirably since the movement of the electrode tool toward the workpiece and the extended movement of the tool result in short circuits during machining. This short circuit and arcing intensify the discharge energy at a high level, and high energy deteriorates the surface quality through uneven erosion of the work material and tool^40^. In the present Maglev EDM, the material is removed from the workpiece in the form of circular cavities, as shown in Fig. 9. Under the recast layer, the heat penetration is prolonged on the machining surface, and a heated zone is formed, which is called a heat-affected zone (HAZ). Here, the surface morphology was detected by a Zygo new view 9000 3D optical profilometer and an OLYMPUS BX51 M optical microscope. From the obtained images, the presence of the HAZ is evident, as shown in Fig. 9.

During the EDM process, the temperature of the HAZ does not reach the melting point or vaporization stage; hence, the heated material does not detach from the location. However, the properties of the microstructure change. Since the produced discharge energy is shared by each element of the process, namely, the workpiece, dielectric medium, and tool, a HAZ is formed in both electrodes^41^. The refinement of the machined surface by a profilometer gives more accurate results and clearly shows the HAZ during the formation of the desired cavity of 2 mm. Moreover, in the present investigations, the natural flushing technique is applied; therefore, the accumulation of unwanted debris can be observed on the workpiece surface. This debris can be washed out or minimized using forced pressure flushing and by employing a fresh dielectric medium. During the Maglev EDM, some micro crakes are often seen in the workpiece surface of the thermal shocks and quenching of the newly machined surface.

**Figure 10:**
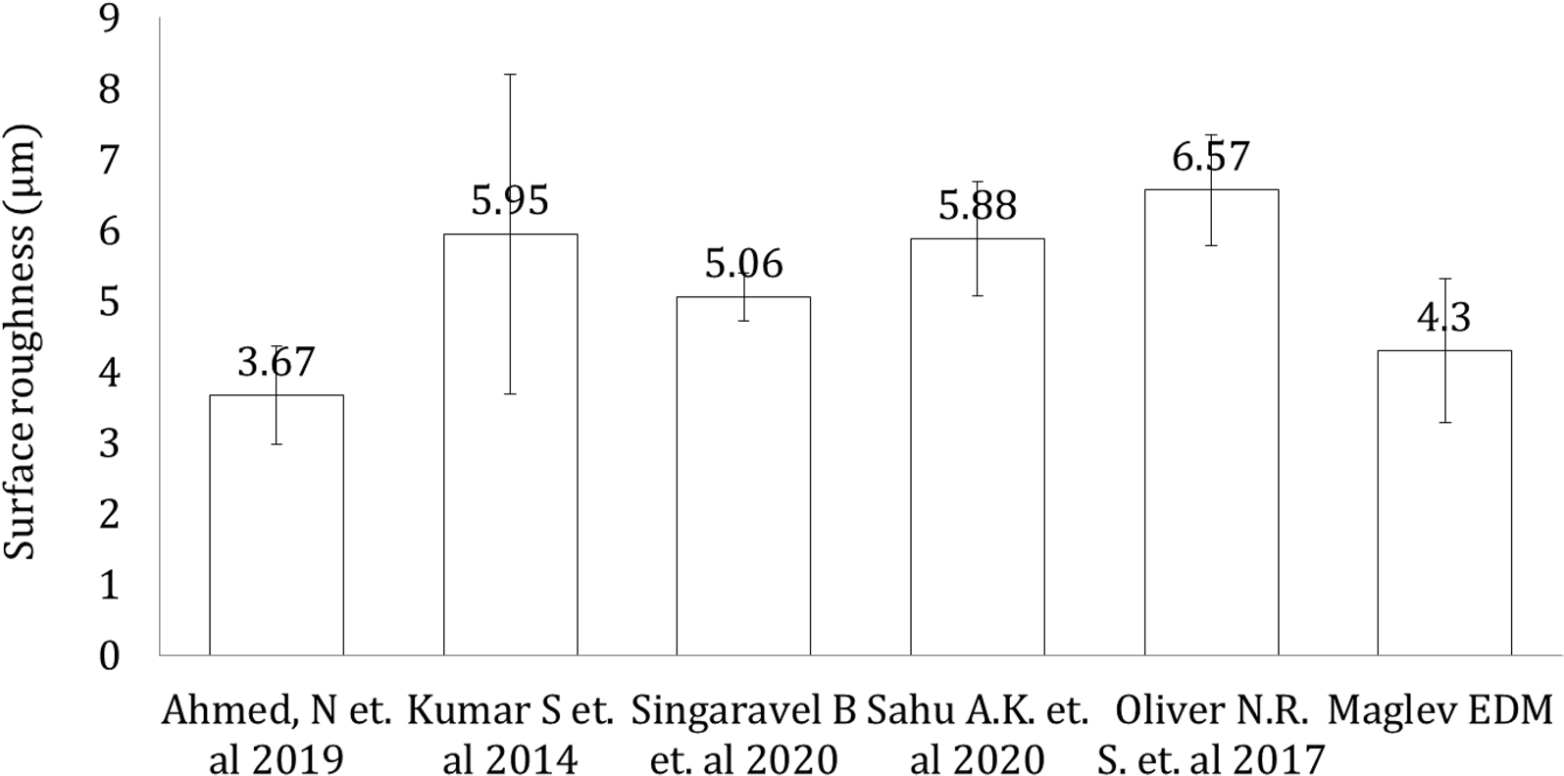
Comparison of the surface roughness.

The characterization of the surface morphology is more volatile since the specific energy density is primarily affected by the current–voltage characteristics and the gap condition of the electrodes. In the present Maglev EDM, the waveforms and gap conditions are maintained to ensure a high surface finish with minimum loss of pulse discharge energy. In Maglev EDM, the primary value of the surface roughness varies from 3.08 to 5.17 μm, and the average surface roughness is obtained at 4.085 μm. In Fig. 10, the average surface roughness of the Maglev EDM is presented and compared with other micro EDM processes that have similar MRR ranges. It has been observed that the range of the average surface roughness is lower in Maglev EDM compared with other conventional EDMs. The average surface roughness in the current work was in the range of 4.3 μm, which was slightly higher than the surface roughness obtained by Ahmed et al.^7^, which was 3.67 μm. They employed a forced flushing technique to carry out the debris and eroded metal particles, whereas in the case of the current Maglev EDM, natural flushing was applied. However, other researchers have determined various ranges of the surface roughness, such as Kumar et al.^42^, who obtained an average surface roughness in the range of 5.95 μm. They used the hybrid Taguchi-artificial neural network approach to predict the surface roughness under different conditions.

The average surface roughness observed by Singaravel et al.^43^ was in the range of 5.06 μm. They tested and concluded that vegetable oil did not function as a dielectric medium in a few cases and enhanced the surface roughness compared to other dielectric media. Sahu et al.^44^ observed an average surface roughness in the range of 5.88 μm during comparative experiments of various types of tools. The average surface roughness observed by the Oliver et al.^45^ was in the range of 6.57 μm. They proposed optimum process parameters to attend to a lower surface roughness index. The research that contributed to the field of EDM of hard-to-cut materials pointed out that the peak current is the most significant parameter and leads to unwanted material removal during EDM. The pulse on time is the second factor that affects the surface roughness. Both parameters ultimately increase the specific energy density while machining and affect the mechanism of the material removal rate.

## Conclusions

The novel Maglev EDM is explored by experiments on the CP-TI workpiece using the brass electrode. The results revealed that the proficiency of the Maglev EDM is higher than that of the conventional EDM. The material removal rate, specific energy and surface morphology were studied as output responses and compared with the available literature. The following conclusions are drawn from the current work:

1. MRR is higher in Maglev EDM than in conventional micro EDM due to the near-zero pulse off time. This relationship increases the desired discharge energy throughout a longer period.
2. The IEG is precisely controlled and regulated by the maglev servo mechanism as per the requirement of the EDM. The spark gap is accurately maintained by the logical arrangement of the magnets using the Maglev lucidity and replacing them with a conventional servo mechanism.
3. The short circuit is the prime concern in conventional micro EDM; short circuits deteriorate the surface quality and increase the tool wear rate. At present, Maglev EDM has resolved such issues to a great extent.
4. Maglev EDMs have shown less specific energy than other conventional EDMs by replacing the conventional pulsed power supply and effective utilization of maximum energy.

The above EDM assessment reveals that the novel Maglev EDM is more proficient and feasible than the conventional micro EDM, which uses a traditional power supply and servomechanism. Moreover, the present novel Maglev EDM is in an early stage, and it is expected that further improvement in the current machine will tremendously enhance the process outputs.

### Future scope

The present developed technology and the prototype are in their early stages. Hard and challenging materials such as Ti6Al4V, CPTI, nimonic, duplex steel with different tool materials, and dielectric media (hydrocarbons and gases) are under examination. The results are in line with the current work. Improvements have been noted, and the work will be reported in future publications. It is expected that the reported technology could be a replacement for the existing servo mechanism due to its simplicity, low cost, and low maintenance, as well as the availability of the components.
